# From Reshaped Metabolome to Repaired Skin: Fermented *Gastrodia elata* Alleviates UVB-Induced Damage Through Controlled Immune Activation

**DOI:** 10.3390/antiox15010045

**Published:** 2025-12-29

**Authors:** Xing Huang, Xiaoqi Yang, Chunrui Xu, Jiajia Liu, Yuan Luo, Zixian Xu, Shixiao Pu, Zongyang Li, Yunlong Zhang, Min Bai, Lianbing Lin

**Affiliations:** 1 Faculty of Life Science and Technology, Kunming University of Science and Technology, Kunming 650500, China; hx0802315@163.com (X.H.); yangxiaoqi991@163.com (X.Y.); xuchunrui890@163.com (C.X.); liujiajialjj@163.com (J.L.); lyuany202507@163.com (Y.L.); xuzx8463@163.com (Z.X.); 15126006206@163.com (S.P.); onmymountainsides@gmail.com (Z.L.); 15974849114@163.com (Y.Z.); 17387403091@163.com (M.B.); 2 Engineering Research Center for Replacement Technology of Feed Antibiotics of Yunnan College, Kunming 650500, China

**Keywords:** fermented *Gastrodia elata*, skin damage, oxidative stress, inflammatory immune homeostasis

## Abstract

UVB radiation induces cutaneous damage through oxidative stress and immune dysregulation. This study investigated the therapeutic potential of *Gastrodia elata* fermented by *Lactobacillus salivarius* AACE1 (GL) in a mouse model of UVB-induced skin injury. Results demonstrated that GL treatment significantly improved skin morphology, enhanced antioxidant activities (SOD and GSH), reduced oxidative damage (MDA), and balanced inflammatory mediators by upregulating TGF-β and IL-10 while downregulating TNF-α, IL-6, and IL-1β. Transcriptomic analysis revealed that GL specifically activated NOD-like receptor signaling pathway components (*Nlrp3*, *Casp4*, and *Gbp2/5*) while inducing *Tnfaip3* to establish negative feedback control. Metabolomic profiling confirmed that fermentation transformed the metabolite landscape, enriching collagen-related dipeptides, antimicrobial/anti-inflammatory metabolites, and antioxidant cofactors. Importantly, comparative analysis showed that GL is more effective than vitamin E in coordinating multiple signaling pathways and maintaining inflammatory homeostasis. These findings establish GL as an effective natural product that alleviates UVB-induced skin damage through synchronized metabolic remodeling and controlled immune activation.

## 1. Introduction

Ultraviolet B (UVB, 280–320 nm) is a primary exogenous risk factor for skin photodamage, inflammatory responses, and accelerated skin aging [1,2]. UVB can directly damage DNA and induce excessive production of reactive oxygen species (ROS), leading to lipid peroxidation, collagen degradation, and disruption of the skin barrier [3]. In the early stages of stress, the body rapidly activates inflammatory responses, releasing pro-inflammatory cytokines to initiate tissue-protective responses. However, excessive or persistent inflammatory responses can block the healing process and lead to chronic tissue damage [4].

Natural medicines have shown significant potential for tissue damage repair due to their high safety and multi-target regulatory properties [5]. *Gastrodia elata* (*G. elata*) is an edible and medicinal plant with antioxidant [6], anti-inflammatory [7], and neuroprotective properties [8]. However, the low bioavailability of its main active components, such as gastrodin and polysaccharides, limits its medicinal efficacy. *Lactobacillus salivarius* (*L. salivarius*) is a probiotic strain widely used in intestinal immune regulation, which can achieve tissue protection by regulating the body’s immune defense ability and the NF-κB inflammatory pathway [9].

Microbial fermentation is a biologically processed method with a long history that is widely applied in pharmaceutical manufacturing, food processing, and agriculture. Research has confirmed that microbial fermentation, as a biotransformation process, can effectively alter the biological activity of traditional Chinese medicine and food plants, thereby generating new therapeutic effects, including beneficial effects on the composition of the gut microbiota and immune system [10,11,12].

Recent studies have found that microbially fermented *G. elata* exhibits both antidepressant and anti-insomnia effects [13]. Furthermore, research results also indicate that fermented *G. elata* reduces the number of apoptotic cells and regulates the expression levels of various genes involved in neuronal differentiation and DNA repair [12].

However, there are currently no reports on the ability of Lactobacillus-fermented *G. elata* to alleviate UVB-induced skin damage. In addition, our preliminary experimental results indicate that, in addition to possessing antioxidant potential GL can also alleviate skin inflammation and promote tissue repair by reshaping the inflammatory signaling network and balancing the immune and repair processes. Given these considerations, it is reasonable to assume that GL has the potential to be an anti-inflammatory and repair agent for the skin.

This study aims to investigate whether GL alleviates UVB-induced skin damage. To verify this effect, we systematically integrated oxidative stress markers, antioxidant enzymes, inflammatory factors, histopathology (H&E and Masson staining), apoptosis staining (TUNEL), and skin transcriptome analysis in mouse skin to explore the necessary regulation triggered by GL.

## 2. Materials and Methods

### 2.1. Bacterial Strain Source

*L. salivarius* AACE1 was preserved in the China General Microbiological Culture Collection Center (CGMCC), with the preservation number CGMCC No. 20700.

### 2.2. L. salivarius AACE1 Fermented G. elata

Fresh *G. elata* was collected at a planting base in Xiaocaoba Town, Zhaotong City, Yunnan Province (27.78° N, 104.60° E) in 2024. Fresh *G. elata* was washed and chopped into pieces, and mixed in a ratio of gastrodia tuber to sterile water of 1:4 (*w*/*w*). The mixture was processed in a blender (20,000 rpm) for 7 min to obtain a uniform suspension. The suspension was pasteurized (sterilized at 60 °C for 30 min), cooled to room temperature, and inoculated with *L. salivarius* AACE1 at 1.0 × 10^8^ CFU/mL under sterile conditions, with an inoculation ratio of 1:20 (*w*/*w*). The mixture was incubated in a sealed container at 37 °C for 7 days. After fermentation was terminated, the mixture was sterilized at 60 °C for 30 min [14]. After cooling to room temperature, the mixture was centrifuged at 4000 rpm for 5 min to remove the residue. Finally, the product was packaged and quenched with liquid nitrogen to obtain the final GL product.

### 2.3. Animals and Therapy

**Figure 1:**
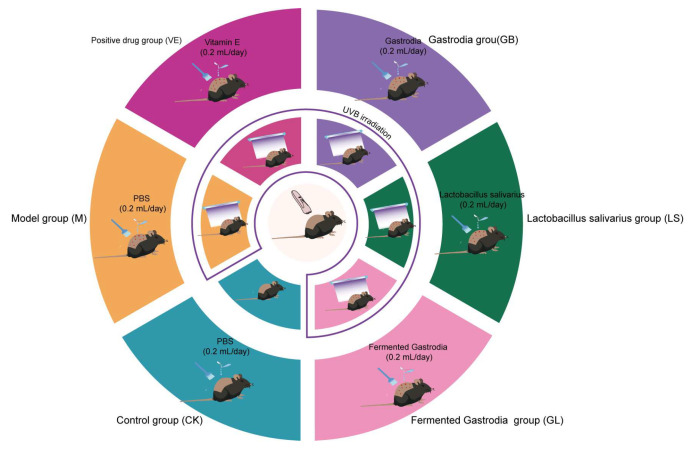
Model construction and group assignment. The innermost circle represents hair removal treatment before modeling. The second circle represents the modeling process, where the purple boxes represent the groups that require UVB irradiation, and the Control groups outside the boxes represent the groups that do not require UVB irradiation. The outermost circle represents the details of back administration, including dosage and type of administration. The classification of each group is placed before the corresponding color.

The 5-week-old C57BL/6J mice were provided by SPF (Biotechnology Co., Ltd., Beijing, China). The housing conditions for the mice were as follows: temperature (22 ± 2 °C), lighting (12-h light/dark cycle), humidity (50–60%), and feeding (a commercial standard laboratory diet with free access to water). All experimental procedures were approved by the Experimental Animal Ethics Committee of the Kunming University of Science and Technology (PZWH (Dian) K2023-0007). After a 1-week acclimation period, mice were randomly divided into six groups (n = 9), as shown in Figure 1. All experimental groups (CK, M, GL, GB, LS, and VE) received topical treatment. The corresponding preparation (0.2 mL PBS buffer for the CK and M groups) was applied evenly to the UVB irradiation area on the shaved backs of the mice daily and gently massaged to ensure absorption. During the application period, individual feeding and observation effectively prevented the mice from licking one another. Three mice from each group were sacrificed on the 5th and 18th days to collect back skin tissue. The tissue samples were immediately frozen in liquid nitrogen and stored at −80 °C for subsequent analysis. The UVB irradiation model was based on our previous research [15], with the following specific parameters: two 15 W UVB lamps (peak wavelength: 313 nm) were used to deliver a calibrated irradiance of 1.5 mW/cm^2^ at the exposure plane. Irradiation was performed for three consecutive days, with daily exposure durations of 50, 50, and 43.3 s, respectively, resulting in a cumulative dose not exceeding 430 mJ/cm^2^. Clinical trial number: not applicable.

According to the disease activity index (DAI) score standard (Table S1) [16], 15 researchers who did not participate in this trial systematically evaluated and recorded the daily macroscopic clinical manifestations of the mice’s backs to ensure the objectivity of the results.

### 2.4. Enzyme-Linked Immunosorbent Assay

The concentrations of malondialdehyde (MDA), superoxide dismutase (SOD), glutathione (GSH), myeloperoxidase (MPO), TGF-β, IL-10, IL-1β, IL-6, and TNF-α in skin tissues were determined using commercially available ELISA kits (spbio, Wuhan, China) according to the manufacturer’s instructions.

### 2.5. Histochemistry

The collected skin tissues were fixed with 4% buffered formaldehyde, embedded in paraffin, stained with hematoxylin and eosin (H&E), Masson’s trichrome, and TUNEL, and then observed under a microscope (Nikon Eclipse E100) (Nikon, Tokyo, Japan). Image acquisition was performed using a PANNORAMIC DESK/MIDI/250/1000 (3DHISTECH, Budapest, Hungary) scanner. Digital sections after H&E and Masson staining were analyzed using CaseViewer 2.4 software, and TUNEL staining was performed using Saiviewer 2.2.4 (Servicebio, Wuhan, China)for image analysis.

### 2.6. Concentration of Endotoxin in GL

Endotoxin levels in the samples were quantified using a commercial Limulus Amebocyte Lysate (LAL) assay kit (rapid gel-clot method; Bioendo, Xiamen, China) according to the manufacturer’s instructions. Briefly, 0.5 mL of the prepared GL product (Section 2.2) was pipetted into a sample test tube (SPL tube). Then, 0.25 mL of the SPL solution was transferred to a positive product control tube (PPC tube). Both tubes were gently mixed and incubated at 37 °C for 21 min in a dry bath incubator. Result Interpretation: A test was considered valid only if the PPC tube exhibited a firm, non-sliding gel upon 180° inversion (+). For valid tests, if the SPL tube showed no gel formation or an incomplete, sliding gel (–), the endotoxin concentration was recorded as <0.25 EU/mL; if the SPL tube formed a firm, stable gel (+), the endotoxin concentration was recorded as ≥0.25 EU/mL.

### 2.7. Metabolomics of G. elata

This study investigated the metabolic alterations in fermented *G. elata* using an untargeted metabolomics approach integrating chromatography (LC) and mass spectrometry (MS). The sample preparation procedures are detailed in the Supplementary Materials. Briefly, plant samples were extracted using a pre-cooled methanol/acetonitrile/water solution (2:2:1, *v*/*v*/*v*, containing 5 ppm L-2-chlorophenylalanine as an internal standard). The extraction process included tissue homogenization, ultrasonication, low-temperature precipitation, and high-speed centrifugation. The supernatant was concentrated to dryness under vacuum. The dried metabolites were reconstituted, filtered through a 0.22 μm membrane, and prepared for LC-MS analysis. The specific reagents and instruments employed in this study are listed in Tables S2 and S3 of the Supplementary Materials, respectively.

Chromatographic separation was achieved using an ACQUITY UPLC HSS T3 column maintained at 40 °C. The mobile phase consisted of 0.1% formic acid in water (phase A) and 0.1% formic acid in acetonitrile (phase B), with the elution gradient program detailed in Table S4 in the Supplementary Materials. Mass spectrometric analysis was performed on a Thermo Orbitrap Exploris 120 mass spectrometer (Thermo Fisher Scientific, Waltham, MA, USA) operated in positive/negative ion switching mode with data-dependent acquisition (DDA) to ensure comprehensive coverage of metabolite profiles.

Raw data were processed using Compound Discoverer™ 3.3 software for peak detection, alignment, normalization, and missing value imputation. Metabolite identification was conducted by searching public databases, including mzCloud, HMDB, and Lipid MAPS. Differentially expressed metabolites between the experimental groups were identified through integrated multivariate statistical analysis and univariate tests (Student’s *t*-test, fold-change), followed by KEGG pathway enrichment analysis to elucidate their biological significance. The data generated in this study have been saved in the NGDC (https://ngdc.cncb.ac.cn/ (accessed on 15 October 2025)) and can be freely obtained, upload number: subPRO072086, project number: PRJCA049127.

### 2.8. Mice Skin Transcriptome

Trizol reagent (Invitrogen, Carlsbad, CA, USA) was used to extract total RNA from the mouse skin, and the concentration and purity were detected using a Nanodrop (Thermo Scientific, Waltham, MA, USA), and the integrity was detected using an Agilent 2100 (Agilent Technologies Inc., Santa Clara, CA, USA). Select ≥ 1 μg RNA, and use the NEBNext Ultra II strand-specific library building kit (New England Biolabs Inc., Ipswich, MA, USA) to build a library. The mRNA was enriched by magnetic beads, interrupted, cDNA was synthesized, the ends were repaired, the linker ligated, fragments screened, and PCR amplified. The quality and concentration of the library were determined using an Agilent 2100 (Agilent) and PicoGreen (Invitrogen), and the same amount was mixed after qPCR quantification. Finally, the Illumina platform PE150 mode was used for sequencing the libraries. The sequences generated in the present study were deposited in NCBI (https://www.ncbi.nlm.nih.gov/ (accessed on 10 October 2025)) and are available under the accession number SUB15637225.

### 2.9. Differential Gene Expression and Functional Enrichment Analysis

Differential gene expression was analyzed using DESeq2 (v1.38.3). Principal component analysis (PCA), correlation analysis heat map, volcano map, and Venn map were all created using the free online platform Personalbio GenesCloud. The significant pathway factor map and differential gene heat map were drawn using https://www.bioinformatics.com.cn/ (accessed on 17 October 2025), using ChiPlot (https://www.chiplot.online/) (accessed on 20 October 2025) to draw a heat map of the correlation between significant metabolites and physico-chemical factors.

### 2.10. Statistical Analysis

Data analysis was performed using IBM SPSS Statistics (version R27.0.1.0) (IBM Corp., Armonk, NY, USA). The Shapiro-Wilk test was used to evaluate normality. Homogeneity of variance was confirmed using Levene’s test. One-way ANOVA and Tukey’s post hoc tests were used for multiple group comparisons. Line plots, histograms, and violin plots were created using GraphPad Prism 8 (GraphPad Software, San Diego, CA, USA). Significance threshold: *p* < 0.05.

## 3. Results

### 3.1. GL Alleviates UVB-Induced Acute Skin Damage in Mice

**Figure 2:**
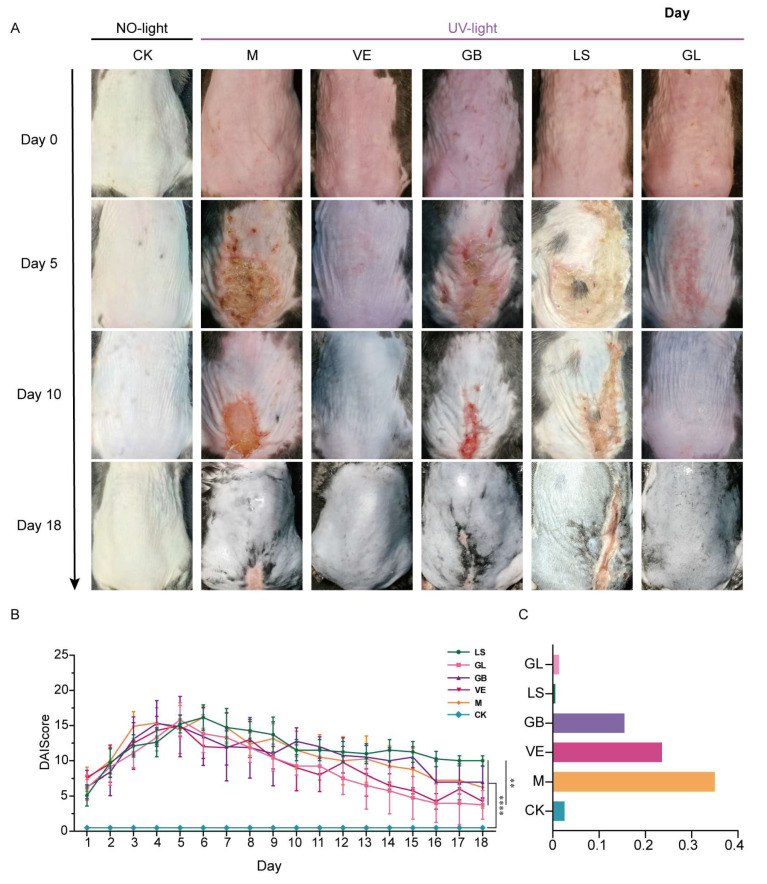
Skin condition of mice. (**A**) On days 0, 5, 10, and 18, the visual field of the back skin in each group (from top to bottom, the apparent changes in the back skin of the same group and mouse at different times). (**B**) DAI score of mice dorsal skin, ** *p* < 0.01, **** *p* < 0.0001, n = 9. (**C**) TUNEL-stained red positive cells in each group on day 18 (%).

**Figure 3:**
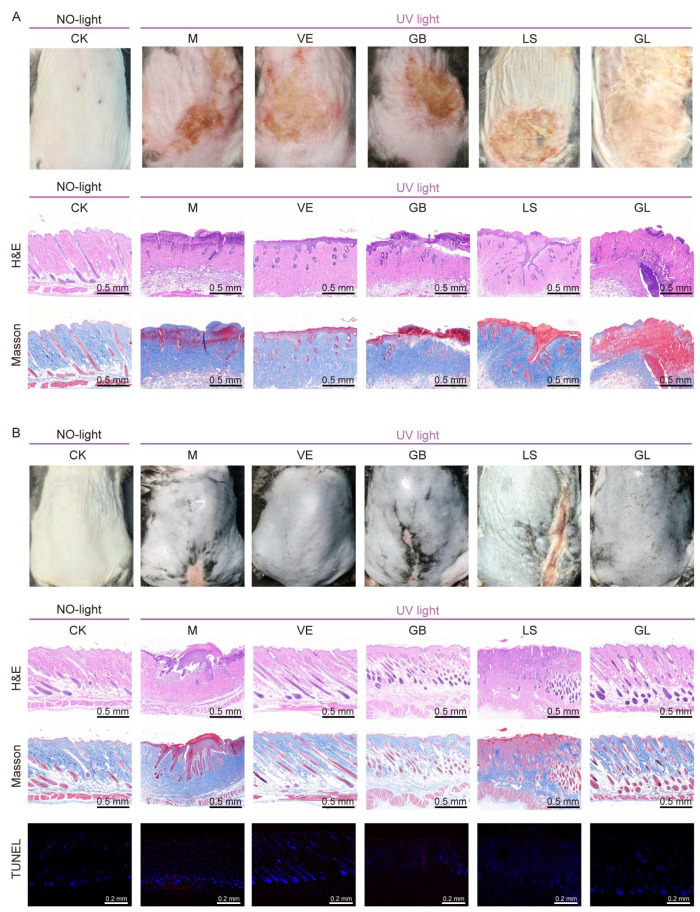
Histopathological analysis and apoptosis of mice dorsal skin (**A**) On day 5, the visual field of the mice’s back used for staining, H&E, and Masson staining pictures of each group (**B**) The visual field of the mice’s back used for staining on day 18, H&E, Masson, and TUNEL staining pictures of each group. Dorsal photographs in Figure 3B are provided for direct macroscopic correlation with the underlying histology. The Day 5 image (**B**, top) is from a parallel cohort sacrificed for initial sampling (different from the longitudinal mouse shown in Figure 2A). The Day 18 image (**B**, top) shows the same mouse as in Figure 2 at the endpoint, linking its final appearance to the histological sections (**B**, bottom) from the same animal.

The macroscopic results for the mice are shown in Figure 2A. The results showed that all experimental groups (M, VE, GB, LS, and GL) exhibited acute light damage characteristics after UVB irradiation, including skin thickening, wrinkling, erythema, and edema (Figure 2A, day 0). The degree of skin damage peaked from the 4th to the 6th day after irradiation, and the corresponding DAI score was the highest (Figure 2B). On the 10th day, the recovery of the GL group was similar to that of the VE group and better than that of the GB and M groups (Figure 2A, day 10). By the 18th day, the skin lesions in the GL and VE groups had disappeared, while obvious lesions were still present in the M, GB, and LS groups (Figure 2A, day 18). In general, with the extension of time, the skin condition of each group gradually improved, and GL and VE treatment significantly promoted the repair of UVB-induced skin damage. Furthermore, the results of skin histological analysis showed that on the 5th day of the experiment, H&E and Masson staining showed that, except for the CK group, the other groups presented typical early features of skin damage and repair, including incomplete epidermal structure, dermal necrosis, inflammatory cell infiltration, and loose, irregular, and light staining of collagen fibers in granulation tissue (Figure 3A). By the 18th day, the two staining results revealed significant differentiation of the repair effect in each group: the M, GB, and LS groups had incomplete recovery, and there were still pathological phenomena such as acanthosis, abnormal hyperplasia, and continuous inflammatory infiltration, respectively. Although the collagen fibers showed a small to large number of proliferations, they were mostly thin and immature; in contrast, the GL and VE groups showed the best comprehensive repair effect. The skin epidermal structure was intact, the cell morphology and arrangement returned to normal, and the dermal structure was uniform. Moreover, the GL group exhibited prominent collagen repair. A large number of mature collagen fibers with a regular arrangement and close to normal levels were observed in the dermal layer of the GL group, and the overall effect was better than that of the VE group (Figure 3B). Moreover, we performed TUNEL staining on the skin of mice on day 18. We synthesized the TUNEL staining results (Figure 3B), the average optical density value chart (Figure 2C), and the skin appearance after treatment in different groups. The results showed that the apoptosis rate of the M group was 35.02%, and the apparent repair state was poor; The apoptosis rate of the GB group was 15.49%, and the apparent repair was still poor; The apoptosis rate of the LS group was only 0.54%, but the apparent repair was lagging be-hind; While the GL group maintained a low apoptosis rate (1.36%), the apparent repair was good.

### 3.2. Dominant Performance of GL in Oxidative Stress and Inflammation Relief

**Figure 4:**
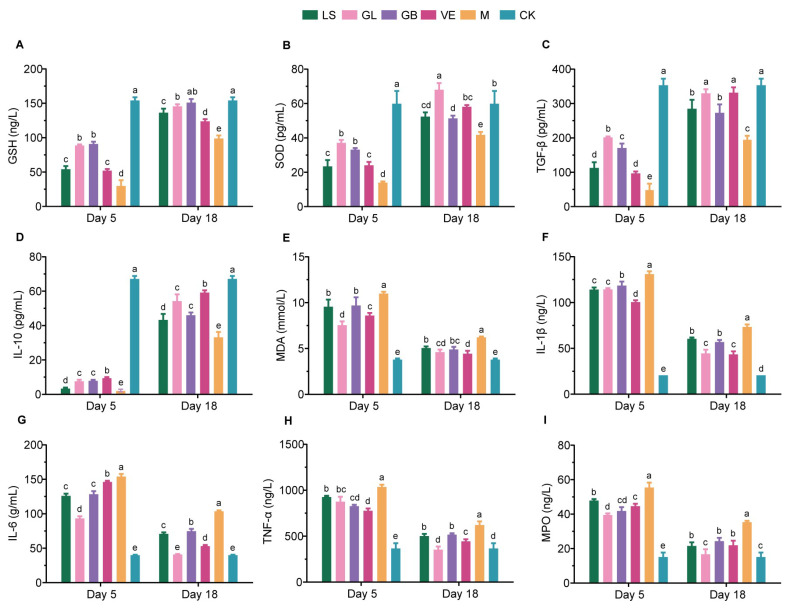
Effects of GL on physiological Effects of GL on physiological and biochemical indices of mice. (**A**) GSH. (**B**) SOD. (**C**) TGF-β. (**D**) IL-10. (**E**) MDA. (**F**) IL-1β. (**G**) IL-6. (**H**) TNF-α. (**I**) MPO. The same letter indicates no significant difference between the two groups (*p* > 0.05), while different letters indicate significant differences (*p* < 0.05).

With the advancement of the experimental process, the skin antioxidant factors (GSH and SOD) in UVB-treated mice showed an overall downward trend. The level of the oxidative damage marker MDA decreased, pro-inflammatory factors (IL-6, IL-1β, TNF-α, and MPO) decreased, and anti-inflammatory factors (TGF-β and IL-10) increased (Figure 4). Group M was in a state of oxidative stress and inflammatory response on both days 5 and 18, with significantly lower levels of GSH, SOD, TGF-β, and IL-10 compared to Group CK (*p* < 0.05), while the levels of IL-6, IL-1β, TNF-α, MDA, and MPO were significantly higher (*p* < 0.05).

On the 5th day, the GL group exhibited the most prominent antioxidant and anti-inflammatory effects. In terms of oxidative indices, the GSH and SOD levels in the GL and GB groups were significantly higher than those in the M, LS, and VE groups (*p* < 0.05) and were close to those in the CK group. The MDA level in the GL group was significantly lower than that in the other UVB-treated groups (*p* < 0.05). In terms of inflammatory factors, the levels of IL-1β and TNF-α in the GL and GB groups were not significantly different (*p* > 0.05) but were significantly lower than those in the M group (*p* < 0.05). The levels of IL-6 and MPO in the GL group were the lowest among all treatment groups, significantly lower than those in the other groups (*p* < 0.05), and closest to those in the CK group. The levels of IL-10 in the GL and GB groups were similar (*p* > 0.05) and lower than those in the VE and CK groups. The level of TGF-β in the GL group was significantly higher than that in the other treatment groups (*p* < 0.05) and was closest to that in the CK group. These results indicate that the GL group effectively alleviated oxidative stress and inflammatory damage on the 5th day, during the peak period of inflammation.

On the 18th day, the recovery effect in the GL group was more significant. In terms of oxidative indices, the GSH level in the GL group was still consistent with that in the GB group (*p* > 0.05). The SOD level was significantly higher than that of the CK group and other treatment groups (*p* < 0.05). The level of MDA in the GL group was similar to that in the VE group (*p* > 0.05) and only slightly higher than that in the CK group (*p* < 0.05). In terms of inflammation, the levels of IL-6, TNF-α, and MPO in the GL group were restored to the same level as those in the CK group (*p* > 0.05) and were significantly lower than those in the other treatment groups (*p* < 0.05). The level of IL-1β was similar to that of the VE group (*p* > 0.05), which was the closest to that of the CK group in all groups. The level of IL-10 was second only to that of the VE and CK groups (*p* < 0.05); the level of TGF-β had reached the level of the VE and the CK group, with no significant difference (*p* > 0.05).

### 3.3. Endotoxin Test Results

The endotoxin test results showed that PPC was positive, SPL was negative, and the endotoxin concentration in the sample was less than 0.25 EU/mL. Please refer to Figure S1 for further details.

### 3.4. Fermentation Conditions and Metabolomics

**Figure 5:**
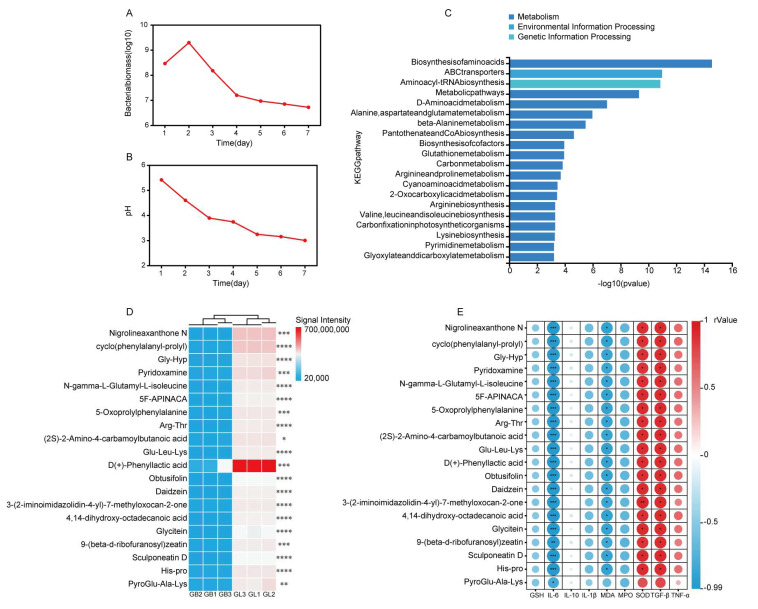
Untargeted metabolomics. (**A**) Changes in bacterial biomass during fermentation. (**B**) Changes in pH during fermentation. (**C**) KEGG enrichment analysis results of differentially expressed metabolites (top 20). (**D**) Signal value heatmap of differentially expressed metabolites in the GL and GB groups(|log2FoldChange|, top 20). (**E**) Correlation heatmap of significantly differentially expressed metabolites and physicochemical factors. For one of these metabolites, we use the abbreviation 5F-APINACA to represent its full chemical name: N-(Cyclopropylmethyl)-1-(5-fluoropentyl)-1H-indole-3-carboxamide. * *p* < 0.05, ** *p* < 0.01, *** *p* < 0.001 and **** *p* < 0.0001.

During the 7-day fermentation process, the inoculated *L. salivarius* AACE1 rapidly grew, reproduced, and metabolized to produce acid, causing the pH of *G. elata* to rapidly decrease from the initial value of 5.42 to 3.01 (Figure 5B). Correspondingly, after reaching its peak on the second day (2 × 10^9^ CFU/mL), the number of viable bacteria continued to decrease to 5.32 × 10^6^ CFU/mL due to lactate accumulation and self-inhibition in a low pH environment (Figure 5A).

KEGG pathway enrichment analysis of the differential metabolites revealed that the metabolic alterations in fermented *G. elata* were significantly enriched in several pathways closely linked to their bioactivities. These were primarily concentrated in the biosynthesis and metabolism of amino acids (such as alanine, aspartate, and glutamate metabolism; and arginine and proline metabolism), glutathione metabolism, and beta-alanine metabolism (Figure 5C). Notably, glutathione metabolism is a crucial intracellular antioxidant pathway [17], while the metabolism of amino acids like arginine and proline is directly implicated in skin tissue repair and immunomodulation. Furthermore, pathways such as D-amino acid metabolism and arginine biosynthesis suggest potential antimicrobial and anti-inflammatory activities in the fermented product. These results indicate that fermentation reshapes the metabolic profile of *G. elata*, significantly enhancing its potential antioxidant, anti-inflammatory, and antimicrobial activities, as well as promoting skin repair.

**Figure 6:**
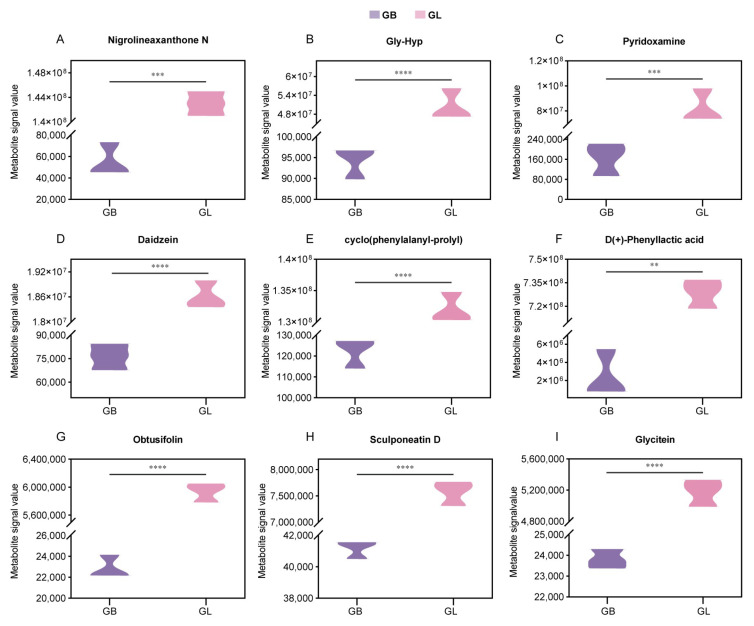
Significant differential metabolites. (**A**–**I**) Violin plot of differential metabolite expression levels related to this study. ** *p* < 0.01, *** *p* < 0.001, **** *p* < 0.0001.

Screening of the top 20 differential metabolites based on |log2FoldChange| revealed that the levels of multiple key bioactive substances in *G. elata* were significantly increased after *L. salivarius* AACE1 fermentation (Figure 5D), and these substances have clear functional potential in skin repair. Among them, Nigrolineaxanthone N (log2FC = 11.34) and Obtusifolin (log2FC = 8.02), promising active molecules (Figure 6A,G), have been reported to possess significant antimicrobial and anti-inflammatory properties [18,19], helping to maintain skin microecological balance and create a favorable environment for repair. Concurrently, fermentation significantly promoted the generation of various functional amino acid derivatives. For instance, Gly-Hyp (log2FC = 9.06) (Figure 6B), which is closely associated with collagen synthesis [20], and the dipeptide cyclo(phenylalanyl-prolyl) (log2FC = 10.08) (Figure 6E), a small cyclic dipeptide that has been reported to readily cross biological membranes and is therefore considered to have potential skin penetration [21]. Furthermore, fermentation specifically enriched D(+)-Phenyllactic acid (log2FC = 8.23) (Figure 6F), which is known for its natural antimicrobial properties [22]. Simultaneously, Sculponeatin D (log2FC = 7.52) (Figure 6H) is an enmein-derived ent-kaurane diterpenoid, and the documented antibacterial activity of enmein against Gram-positive bacteria suggests that Sculponeatin D may exert similar antimicrobial effects [23,24]. Glycine (log2FC = 7.76) (Figure 6I) and Daidzein (log2FC = 7.93) (Figure 6D), which are transdermally absorbable, reduce keratinocyte apoptosis and have antioxidant and anti-inflammatory effects [25,26,27]. Notably, pyridoxamine (log2FC = 8.96) (Figure 6C), a form of vitamin B6, plays a key role in protein metabolism and maintenance of skin health [28,29]. Collectively, these results demonstrate that fermentation treatment can effectively and directionally enhance the potential of *G. elata* to promote collagen production, exert antimicrobial and anti-inflammatory effects, and maintain the skin barrier.

According to the correlation analysis results, the pro-inflammatory cytokine IL-6 was significantly negatively correlated with the target differential metabolites (r < −0.97, *p* < 0.001), and the oxidative damage marker MDA was also significantly negatively correlated with these metabolites (r < −0.85, *p* < 0.05). At the same time, the antioxidant enzyme SOD (r > 0.87, *p* < 0.05) and the immune regulatory factor TGF-β (r > 0.90, *p* < 0.05) showed significant positive correlations with the target metabolite group (Figure 5E and Figure 6A–I). These results indicate that the differential metabolites involved in Figure 6A–I may collectively participate in inhibiting inflammatory responses, reducing oxidative damage, and enhancing the body’s antioxidant capacity and immune regulatory functions.

### 3.5. GL-Mediated Gene Regulation

**Figure 7:**
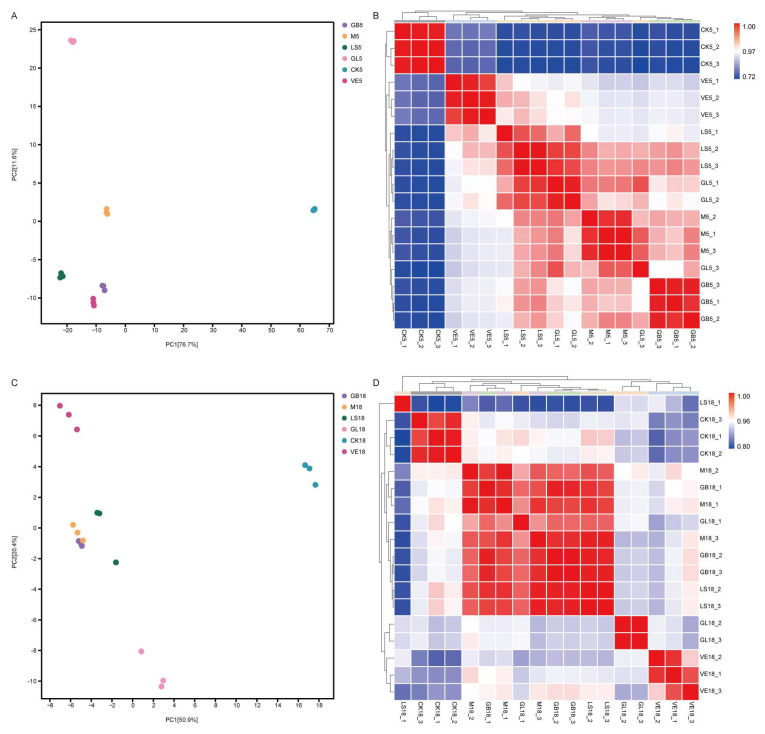
Expression analysis. (**A**) PCA analysis on day 5. (**B**) Correlation analysison day 5. (**C**) PCA analysis on day 18. (**D**) Correlation analysis on day 18.

To reveal the molecular mechanism of GL in the repair of UVB-induced skin damage, we performed transcriptome analysis of the dorsal skin tissueof mice. Gene expression correlation analysis (Figure 7B,D) showed that the experimental data were stable and that the sample grouping was reasonable. Principal component analysis (PCA) (Figure 7A,C) further revealed the obvious separation among the groups, indicating that there were significant differences in their transcript levels.

**Figure 8:**
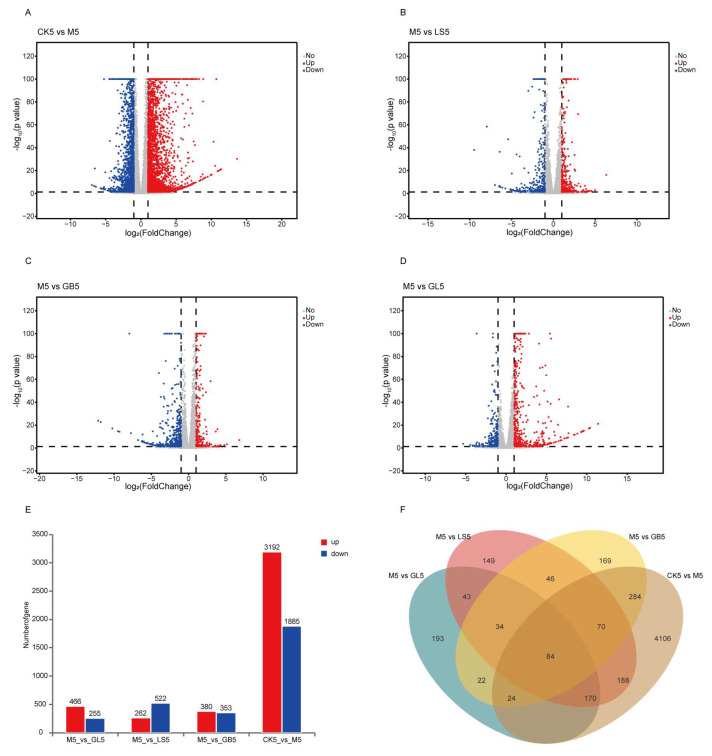
Differential genes on day 5. (**A**) DEG volcano map of the CK and M groups. (**B**) DEG volcano map of groups M and LS. (**C**) DEG volcano diagram of groups M and GB. (**D**) DEG volcano map of groups M and GL. (**E**) Number of DEGs in the CK, M, LS, GB, and GL groups. (**F**) Venn analysis revealed that 84 genes overlapped. Volcano plot: The two vertical dashed lines indicate the fold-change threshold; the horizontal dashed line represents the significance threshold.

**Figure 9:**
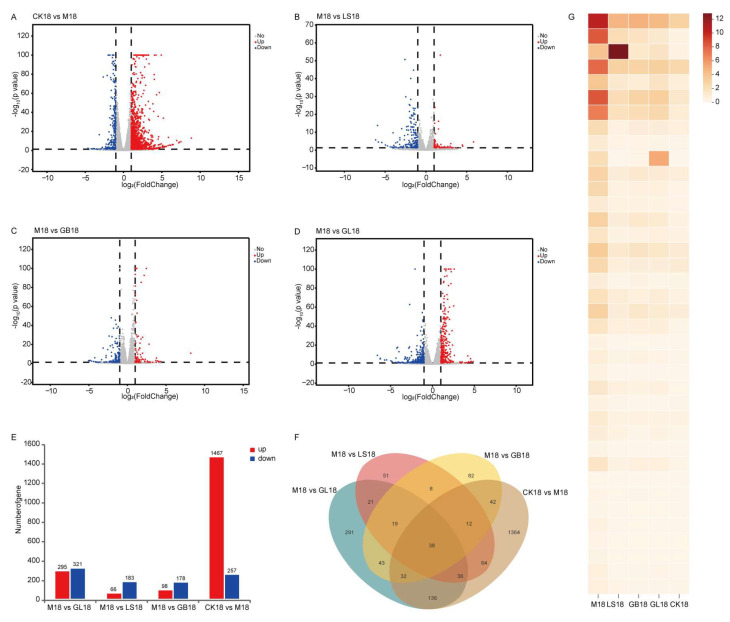
Differential genes on day 18 (**A**) DEG volcano map of the CK and M groups (**B**) DEG volcano map of the M and LS groups. (**C**) DEG volcano diagram of groups M and GB. (**D**) DEG volcano map of group M and group GL. (**E**) The number of DEGs in the CK group, M group, LS group, GB group, and GL group. (**F**) Venn analysis showed that 38 genes overlapped. (**G**) Heatmap of 38 overlapping DEGs. Volcano plot: The two vertical dashed lines indicate the fold-change threshold; the horizontal dashed line represents the significance threshold.

In order to analyze the changes in gene expression, we performed differential gene expression (DEG) analysis and counted the number of genes. On day 5, 5077 differentially expressed genes were identified using skin transcriptome analysis. Compared with the CK group, 3192 genes were upregulated, and 1885 genes were downregulated in the M group. Compared with the M group, 466 genes were upregulated, and 255 genes were downregulated in the GL group; 262 genes were upregulated, and 522 genes were downregulated in the LS group; and in the GB group, 380 genes were upregulated, and 353 genes were downregulated (Figure 8A–E). Venn analysis showed that 84 genes were significantly regulated in the CK group compared with the M group and in the M group compared with the LS, GB, and GL groups (Figure 8F). By day 18, 1724 differentially expressed genes were identified. Compared with the CK group, 1467 genes were upregulated, and 257 genes were downregulated in the M group. Compared with group M, 295 genes were upregulated, and 321 genes were downregulated in group GL; in the LS group, 66 genes were upregulated, and 183 genes were downregulated; in the GB group, 98 genes were upregulated, and 178 genes were downregulated (Figure 9A–E). Venn analysis showed that 38 genes were significantly regulated in the CK group compared with the M group and in the M group compared with the LS, GB, and GL groups (Figure 9F). The heat map of these 38 overlapping DEGs showed that *G. elata* and fermented *G. elata* s reversed the regulation of UVB skin damage and brought the gene expression pattern closer to that of the CK group (Figure 9G). According to the different numbers of DEGs in each group, we found that the regulation range of the GL group was equivalent to that of the GB and LS groups on the 5th day, and the regulation range of the GL group was significantly higher than that of the other UVB treatment groups on the 18th day. The results showed that the regulatory effect of the GL group at the transcriptome level was not only better than that of GB and LS, but also closer to the normal state in the recovery stage.

### 3.6. Mice Skin Differential Gene KEGG Signaling Pathway

**Figure 10:**
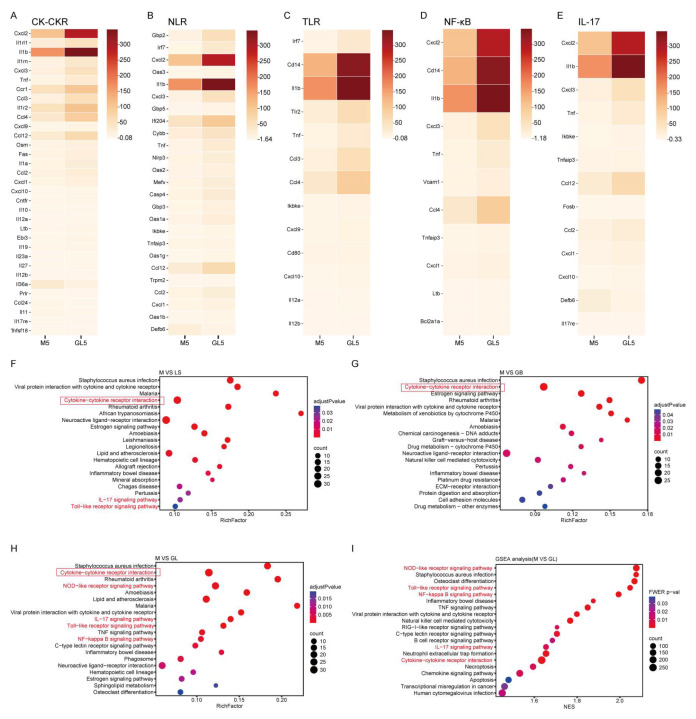
Differential gene expression profiles and their functions. (**A**) Heat map of gene expression related to groups M and GL in the Cytokine-cytokine receptor interaction pathway. (**B**) Heatmap of gene expression related to groups M and GL in the NOD-like receptor signaling pathway. (**C**) Heatmap of gene expression related to groups M and GL in the Toll-like receptor signaling pathway. (**D**) Heatmap of related gene expression in the M and GL groups in the NF-κB signaling pathway. (**E**) Heatmap of gene expression related to groups M and GL in the IL-17 signaling pathway. (**F**) On the 5th day, the pathways were significantly enriched in the M and LS groups. (**G**) On the 5th day, pathways were significantly enriched in the M and GB groups. (**H**) On the 5th day, the pathways were significantly enriched in the M and GL groups. (**I**) GSEA analysis of all genes in the M and GL groups. The circled red box indicates the same content, the red font indicates the pathway we are concerned about.

Because the experimental groups had the most significant skin inflammatory response and the largest number of differentially expressed genes (DEGs) on the 5th day, we focused on analyzing the different regulatory mechanisms of the inflammatory response in the LS, GB, and GL groups at this time point. KEGG enrichment analysis showed that compared with the M group, the inflammation-immune-related pathways in the three treatment groups were enriched to varying degrees, and the Cytokine-cytokine receptor interactions (CK-CKR) pathway was a common upregulated pathway among the three groups (Figure 10F–H). The differentially expressed genes in this pathway mainly include *Cxcl1/2/3/9/10* belonging to the CXC subfamily, *Ccl2/3/4/12* belonging to the CC subfamily, various cytokines such as *Il1a*, *Il1b*, *Il12b*, *Il27*, and *Osm*, as well as *TNF*, *Fas*, and *Ltb* belonging to the TNF family (Figure 10A). At the same time, the GL group showed the strongest enrichment in this pathway (DEGs = 33), indicating stronger regulation characteristics of the inflammatory response.

On this basis, Pattern Recognition Receptors (PRR)-mediated innate immune pathways were significantly upregulated, including the Toll-like receptor signaling pathway (TLR, including *Tlr2*, *Cd14*, *Cd80*,*Il12a/12b*, and other genes) and the NOD-like receptor signaling pathway (NLR, including *Gbp2/3/5*, *Nlrp3*, *Irf7*, *Casp4*, *Mefv*, *Oas1a/1g/3*, *Ifi204*, and other genes) (Figure 10B,C,F,H). Notably, the NLR pathway was significantly enriched only in the GL group, indicating that the GL group not only enhances the recognition of pathogen-associated molecular patterns (PAMPs) but also specifically enhances the perception of damage-associated molecular patterns (DAMPs), thereby jointly activating the formation of inflammasomes and achieving dual recognition and synergistic activation of internal and external danger signals.

Further analysis indicated that the NLR and TLR pathway signals converge to the NF-κB signaling pathway (NF-κB), where related genes such as *TNF*, *Il1b*, *Cxcl/2/3*, *Tnfaip3*, *Bcl2a1a*, and *Vcam1* are significantly upregulated (Figure 10D). At the same time, the inflammatory signals generated by NF-κB are transmitted to the IL-17 signaling pathway (IL-17). In summary, NF-κB acts as an amplifier of inflammatory signals. As a downstream effector pathway, the IL-17 is also continuously activated, with related genes such as *Fosb*, *Cxcl1/2/3/10*, *Ccl2/12*, *Il17re*, *Tnf*, and *Tnfaip3*. (Figure 10E), activating multiple host responses, such as inflammation, host defense, autoimmune, and pro-inflammatory activities (Figure 9).

**Figure 11:**
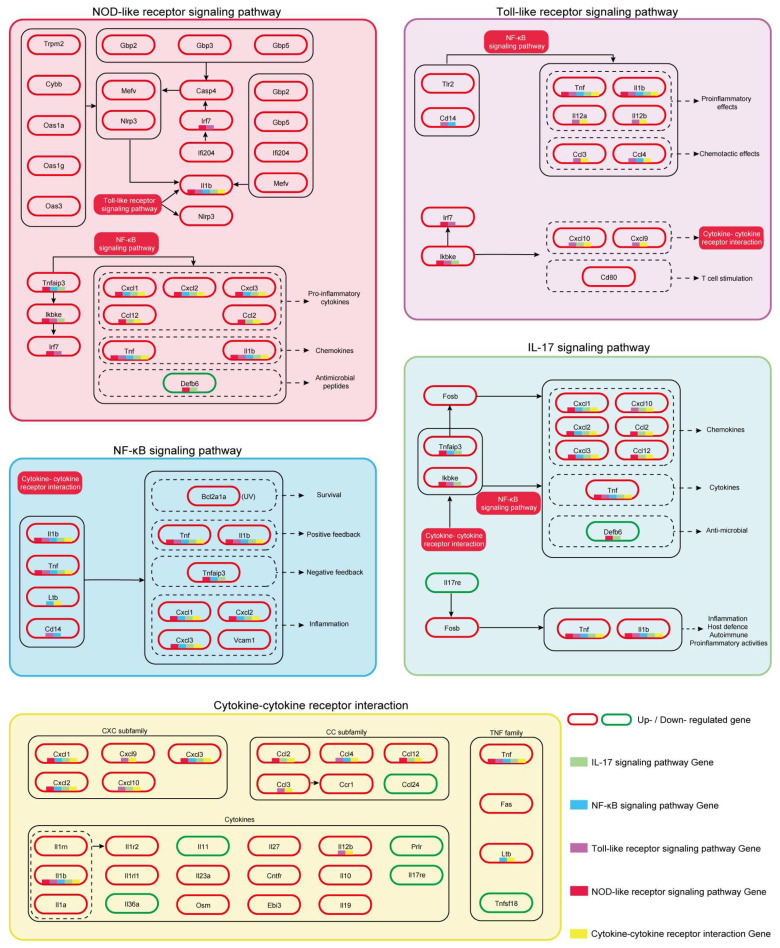
GL-mediated UVB damage repair gene regulatory functional network. The red background and white text in Figure 11 represent the signal pathways connected to it, and different colored backgrounds represent different pathways. The pathway names are labeled at the corresponding positions. The red box and black text represent upregulated genes, and the green box and black text represent downregulated genes. For genes that appeared across multiple pathways, we used the corresponding colors to represent their affiliation, as indicated in the bottom right corner of Figure 11.

To further elucidate gene functions and key molecular pathways, we used two complementary methods to analyze the key pathways of the GL group. Among them, KEGG enrichment analysis takes the list of DEGs as input, while GSEA analyzes the complete list of expressed genes. Figure 10I shows the results of GSEA enrichment analysis based on all genes between groups M and GL (FWER *p* < 0.05, NEW > 1.4, Top 20). The results showed that the NLR, TLR, CK-CKR, NF-κB, and IL-17 pathways were significantly enriched in KEGG and GSEA and were identified as the key driving pathways of *G. elata* fermented by *L. salivarius* AACE1 in the UVB-induced skin damage model. The GL-mediated UVB damage repair gene regulatory functional network is shown in Figure 11.

## 4. Discussion

Excessive exposure of the skin to UVB can lead to oxidative stress and inflammatory reactions, causing skin photoaging and damaging skin tissue structure [30]. Research has shown that UVB irradiation can penetrate the dermis layer, producing ROS in cells and tissues and inducing oxidative stress and the accumulation of the lipid peroxidation product malondialdehyde (MDA), leading to the significant consumption of endogenous antioxidants, such as GSH and SOD in the skin [31,32]. At the molecular level, ultraviolet (UV) radiation activates various pattern recognition receptors (PRRs) in the skin, including Toll-like receptors (*TLRs*) and NOD-like receptors (*NLRs*). Under UV irradiation, keratinocytes and other skin cells upregulate *TLRs*, especially *TLR3*, which can sense UV-induced RNA damage and promote the production of pro-inflammatory cytokines, such as TNF-α and IL-6, thereby triggering acute inflammatory reactions [33]. Meanwhile, ultraviolet radiation can also trigger the activation of NLR family members, such as *NLRP3*, leading to inflammasome assembly, activation of caspase-1, and maturation of IL-1β and IL-18, thereby amplifying local inflammation [34]. Through the joint activation of TLR- and NLR-mediated signaling, ultraviolet radiation triggers a series of innate immune responses, leading to skin inflammation and barrier disruption.

Recent studies have found that active plant ingredients can improve skin damage by regulating the immune-inflammatory network [35,36]. However, most of these components have a single target of action, and their overall regulatory effect on skin damage repair is limited. This study found that the GL component exhibits unique regulatory characteristics through a systematic analysis of macroscopic skin characterization, histopathology, biochemical indicators, and transcriptome data. In the early stages of damage, GL treatment specifically activates the NLR pathway by recognizing PAMPs and DAMPs, causing lysosomal damage and activating the inflammatory response [37]. Among them, the *NLRP3* gene is activated and upregulated. According to previous reports, the *NLRP3* receptor is an important inflammasome complex involved in UV-induced cellular inflammation [38]. It is worth noting that although transcriptome data showed significant activation of multiple inflammation-related pathways, a decrease in pro-inflammatory cytokine levels was observed at the protein level. The difference in gene expression and protein levels may be attributed to the strong antioxidant capacity of GL, which blocks the positive feedback loop of ROS-NF-κB activation [39,40]. The active ingredients of *G. elata* can effectively inhibit the activation of NF-κB.

Of particular note is that this study observed partial integration of the TLR pathway into the NLR pathway, and both pathways ultimately enter the CK-CKR pathway through chemokines and cytokines, forming a complex network of regulation. The NF-κB pathway plays a central role in this network, forming positive feedback by upregulating *TNF* and *Il1b* [41] and achieving negative feedback regulation through *Tnfaip3* [42].

In the later stage of repair, the GL group showed significant tissue repair advantages, especially in terms of the maturity and regularity of collagen fibers, which were significantly better than those in the other groups. This phenomenon is closely related to two mechanisms: firstly, there is an “overcompensation” phenomenon in SOD activity, exceeding normal levels, similar to the results of Khan [43]. After applying the plant alkaloid neferine, UV-induced SOD and GPx activity in the skin of model mice significantly increased, collagen fiber loss was significantly inhibited, and the fiber structure became more uniform; secondly, the sustained high expression of TGF-β provides favorable conditions for tissue reconstruction [44].

Several abnormal phenomena in this study are worth exploring in depth: although the LS group showed the lowest apoptosis rate, the repair effect was significantly delayed; the IL-10 level in the GL group was lower than that in the VE group, but it demonstrated enhanced reparative capacity, which may be due to the fundamental differences in their mechanisms of action. VE mainly exerts its effects through a single antioxidant pathway, while GL, as a composite system with multiple bioactive components, involves a more complex and multi-target regulatory network in its mechanism of action. The inherent complexity of GL enables it to not only suppress inflammation but also balance multiple immune responses, thereby promoting repair. As Cowin et al. [45] pointed out, a specific degree of inflammatory signaling may be necessary for complete repair.

In addition, the untargeted metabolomic comparison between unfermented and fermented *G. elata* further clarifies the mechanistic basis of these effects. Fermentation markedly re-models amino acid–centered pathways, particularly glutathione metabolism and arginine/proline metabolism, which are critically involved in intracellular antioxidant defence and collagen biosynthesis [46,47]. Among the metabolites enriched in fermented *G. elata*, Nigro-lineaxanthone N and obtusifolin have been reported to exert pronounced antibacterial and anti-inflammatory activities, including inhibition of methicillin-resistant *Staphylococcus aureus* and suppression of inflammatory signaling in joint and renal models [18,19,48]. Gly-Hyp is a collagen-derived dipeptide whose hydrolysis by prolidase increases the availability of proline and hydroxyproline, thereby promoting collagen synthesis and matrix regeneration [49]. The membrane-permeable cyclic dipeptide cyclo(phenylalanyl-prolyl), which crosses biological membranes by simple diffusion, along with the broad-spectrum antimicrobial D(+)-phenyllactic acid and enmein-type diterpenoids, indicates an enhanced antibacterial potential of the fermented material [21,50,51]. Furthermore, daidzein, which is transdermally absorbable, has been shown to protect UVB-damaged keratinocytes by scavenging ROS and attenuating oxidative injury [27,52], while pyridoxamine, a natural vitamin B6 vitamer, is a potent inhibitor of advanced glycation and lipoxidation reactions that contribute to tissue damage [29]. Together with the strong negative correlations of these metabolites with IL-6 and MDA and their positive correlations with SOD and TGF-β, these data suggest that fermentation generates a coordinated set of small molecules that collectively reinforce the antioxidant, anti-inflammatory, and reparative actions of fermented *G. elata*.

Compared with traditional antioxidants such as vitamin E (which mainly works through direct free radical scavenging [53,54]), GL exhibits a wider range of effects, including the modulation of multiple pathways and temporal immune regulation. Its unique mode of “activation before adjustment” may be the key to achieving better repair effects. It should be clarified that the comparison between GL and vitamin E in this study aims to reveal the differences in functional output dimensions between complex fermentation systems and single antioxidant molecules rather than to demonstrate mechanistic equivalence. Vitamin E mainly provides electrons to neutralize free radicals through phenolic hydroxyl groups, while GL, as a complex system reshaped by fermentation metabolism, covers multiple dimensions such as antioxidant defense, inflammation signal regulation, antibacterial activity, and accumulation of pro-repair metabolites, reflecting the functional characteristics of two different strategies: ‘system regulation’ and ‘target intervention’. This study did not assume direct comparability between GL and vitamin E in terms of their mechanisms of action, but instead revealed the potential advantages of natural fermentation products in integrating multiple biological activities through functional comparisons, providing a basis for developing skin repair strategies based on multi-target synergy.

Although this study revealed at the systemic level that GL promotes skin repair through a synergistic mechanism involving multiple pathways, the types of immune cells, key receptors, and intracellular signaling nodes directly affected by GL remain unclear. The extent to which the observed immune regulatory effects are directly regulated by GL on immune cells or indirectly reflected in its antioxidant and tissue-protective activities still needs to be further elucidated through targeted experiments at the cellular and molecular levels.

Although early activation of multiple inflammatory pathways by GL did not result in significant adverse reactions, the safety of its multi-pathway activation effect in specific individuals still needs to be carefully evaluated, considering individual differences, especially in the presence of an immune dysregulation background. Future research should focus on exploring the differential response of GL in individuals with different immune states and optimizing drug delivery strategies.

In summary, GL precisely balances immune activation and oxidative damage, regulating the process from innate immune recognition to tissue repair signals at multiple levels, thereby effectively repairing skin injuries and providing a theoretical basis for the development of new skin photoprotection strategies.

### Future Directions

Subsequent research will focus on combining transdermal prediction with in vitro experiments to screen for potential differential metabolite monomers. Using keratinocyte and macrophage models, this study investigated the direct effects of these monomers on key immune pathways, such as *NLRP3* inflammasome activation and NF-κB signaling, in order to clarify the specific substances and mechanisms by which fermented Tianma exerts immune regulatory effects at the molecular level and promotes in-depth research from observation to mechanism. In addition, further exploration will be conducted on the differential response of GL in individuals with different immune states, and its administration mode will be optimized to enhance the specificity and safety of its application.

## 5. Conclusions

**Figure 12:**
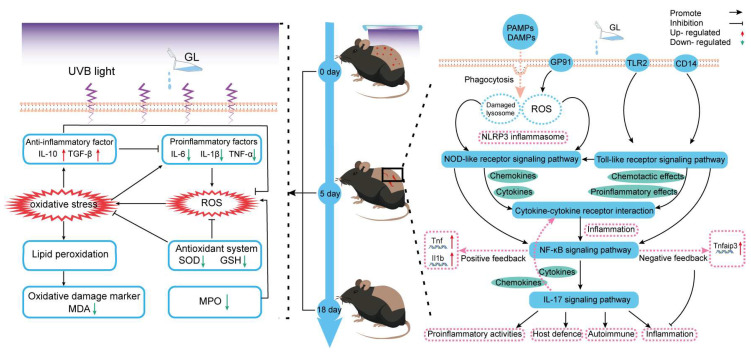
Potential mechanism of GL on UVB-induced skin damage repair in mice. The left side shows the changes in physical and chemical factors and their interrelationships throughout the entire experimental cycle, while the right side shows the interrelationships between signaling pathways activated on the 5th day after administration (during the most severe inflammation period), as well as partial positive and negative feedback regulation.

In summary, this study suggests that GL provides strong protection against UVB-induced skin damage by synergistically promoting structural repair, limiting oxidative damage, and restoring the inflammatory immune balance. The integration of histological, biochemical, transcriptomic, and metabolomic data indicates that fermentation reshapes the metabolome of *G. elata* into an enriched library of collagen-related dipeptides, antibacterial and anti-inflammatory metabolites, and antioxidant cofactors, thereby enhancing their related functions in skin damage repair. Mechanistically, GL accelerates tissue remodeling while maintaining controlled apoptosis, enhances endogenous antioxidant defense, and coordinates TLR/NLR-NF -κB-CK-CKR-IL-17 signaling into a “start amplify restrain” regulatory loop, effectively clearing damage signals without excessive inflammation (Figure 12). These findings provide theoretical support for the development of fermented *G. elata* as a natural agent for photoprotection and tissue repair.

## Data Availability

The untargeted metabolome data generated in this study have been saved in NGDC (https://ngdc.cncb.ac.cn/ (accessed on 15 October 2025)). Free to obtain, upload number: sub-PRO072086, project number: PRJCA049127. The sequences generated in the present study were deposited in NCBI (https://www.ncbi.nlm.nih.gov/ (accessed on 10 October 2025)) and are available under accession number SUB15637225.

## Supplementary Materials

The following supporting information can be downloaded at: https://www.mdpi.com/article/10.3390/antiox15010045/s1, Figure S1: The left side indicates PPC positivity, while the right side indicates SPL negativity. Table S1: DAI coring criteria. Table S2: Reagents. Table S3: Instruments. Table S4. Elution gradient in positive and negative modes.
