# Supplementary material for: From Reshaped Metabolome to Repaired Skin: Fermented Gastrodia elata Alleviates UVB-Induced Damage Through Controlled Immune Activation
Source: Antioxidants (Basel). 2025 Dec 29;15(1):45. doi: 10.3390/antiox15010045 (PMC12837383; doi:10.3390/antiox15010045)
Supplement: Supplementary file 1 [file antioxidants-15-00045-s001.zip › Supplementary material.pdf]

Table S1. DAI coring criteria				
**Index**	**0 points**	**1 points**	**2 points**	**3 points**
Pigmentation	None	Light brown	Medium brown	Dark brown
Scaling	None	Slight	Moderate	Significant
Erythema	None	Mild	Moderate	With edema, crust, and necrosis
Wrinkles	None	Mild	Moderate	Wrinkles
Elasticity	Good	Fair	Poor	Complete loss
Skin thickness	Normal	Mild thickening	Moderate thickening	Marked thickening

Table S2. Reagents				
**Name**	**CAS**	**Purity**	**Brand**	**Part Number**
Methanol	67-56-1	99.9 %	Fisher	A456-4
Acetonitrile	75-05-8	99.9 %	Fisher	A955-4
Formic acid	64-18-6	98.0 %	TCI	F0654
L-2-Chlorophenylalanine	103616-89-3	98.0 %	Aladdin	C105993

Table S3. Instruments		
**Name**	**Brand**	**Model**
Refrigerated centrifuge	Eppendorf	5430 R
Water Purifier	Thermo	Labtower EDI
High-throughput tissue grinder	Jingxin	TL-48R
Ultrasonic cleaning machine	SCIENTZ	SB-800D
Vacuum Concentrator	SCIENTZ	SCIENTZ-1LS
Ultra-high performance liquid chromatography	Thermo	Vanquish Flex
High Resolution Mass Spectrometer	Thermo	Orbitrap Exploris 120

Chromatographic methods

An ACQUITY UPLCHSST3column (100Å, 1.8 μm, 2.1 mm × 100 mm) was used with a flow rate of 0.4 mL/min, a column temperature of 40°C, an autosampler temperature of 8°C, and an injection volume of 2 μL.

Positive and negative mode mobile phase: mobile phase A is 0.1% formic acid water, mobile phase B is acetonitrile (containing 0.1% formic acid), and the elution gradient is as follows:

Table S4. Elution gradient in positive and negative modes

Time（min）	B%
0	5%
1	5%
7	95%
8	95%
8.1	5%
12	5%
