# Supplementary figures and images for: From Reshaped Metabolome to Repaired Skin: Fermented Gastrodia elata Alleviates UVB-Induced Damage Through Controlled Immune Activation
Source: Antioxidants (Basel). 2025 Dec 29;15(1):45. doi: 10.3390/antiox15010045 (PMC12837383; doi:10.3390/antiox15010045)

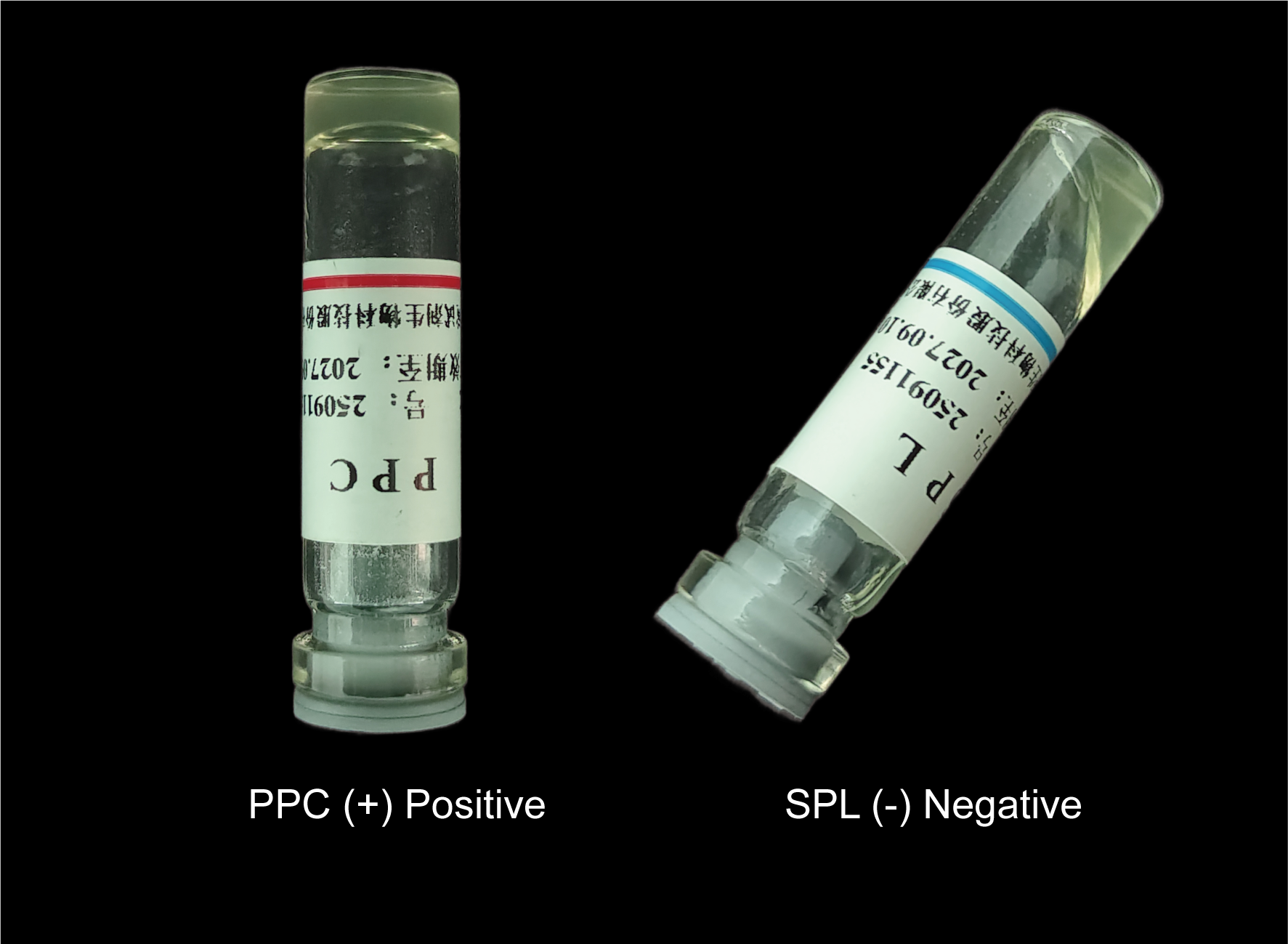

Supplement: Supplementary file 1 [file antioxidants-15-00045-s001.zip › antioxidants-4034756-Figure S1.tif]
